# Clusterin Seals the Ocular Surface Barrier in Mouse Dry Eye

**DOI:** 10.1371/journal.pone.0138958

**Published:** 2015-09-24

**Authors:** Aditi Bauskar, Wendy J. Mack, Jerome Mauris, Pablo Argüeso, Martin Heur, Barbara A. Nagel, Grant R. Kolar, Martin E. Gleave, Takahiro Nakamura, Shigeru Kinoshita, Janet Moradian-Oldak, Noorjahan Panjwani, Stephen C. Pflugfelder, Mark R. Wilson, M. Elizabeth Fini, Shinwu Jeong

**Affiliations:** 1 USC Institute for Genetic Medicine and Graduate Program in Medical Biology, Keck School of Medicine of USC, University of Southern California, Los Angeles, California, United States of America; 2 Southern California Clinical & Translational Science Institute and Department of Preventive Medicine, Keck School of Medicine of USC, University of Southern California, Los Angeles, California, United States of America; 3 The Schepens Eye Research Institute, Massachusetts Eye & Ear and Department of Ophthalmology, Harvard Medical School, Boston, Massachusetts, United States of America; 4 USC Eye Institute, Keck School of Medicine of USC, University of Southern California, Los Angeles, California, United States of America; 5 Research Microscopy and Histology Core, Department of Pathology, Saint Louis University School of Medicine, St Louis, Missouri, United States of America; 6 Department of Pathology and Department of Ophthalmology, Saint Louis University School of Medicine, St. Louis, Missouri, United States of America; 7 The Vancouver Prostate Centre and Department of Urologic Sciences, University of British Columbia, Vancouver, British Columbia, Canada; 8 Department of Frontier Medical Science and Technology for Ophthalmology, Kyoto Prefectural University of Medicine, Kyoto, Japan; 9 Center for Craniofacial Molecular Biology, Division of Biomedical Sciences, University of Southern California, Herman Ostrow School of Dentistry of USC, Los Angeles, California, United States of America; 10 New England Eye Center/Department of Ophthalmology and Department of Developmental, Molecular & Chemical Biology, Tufts University School of Medicine, Boston, Massachusetts, United States of America; 11 Ocular Surface Center, Department of Ophthalmology, Cullen Eye Institute, Baylor College of Medicine, Houston, Texas, United States of America; 12 Illawarra Health and Medical Research Institute, School of Biological Sciences, University of Wollongong, Wollongong, New South Wales, Australia; 13 USC Institute for Genetic Medicine and Departments of Cell & Neurobiology and Ophthalmology, Keck School of Medicine of USC, University of Southern California, Los Angeles, California, United States of America; 14 USC Institute for Genetic Medicine and Department of Ophthalmology, Keck School of Medicine of USC, University of Southern California, Los Angeles, California, United States of America; Cedars-Sinai Medical Center; UCLA School of Medicine, UNITED STATES

## Abstract

Dry eye is a common disorder caused by inadequate hydration of the ocular surface that results in disruption of barrier function. The homeostatic protein clusterin (CLU) is prominent at fluid-tissue interfaces throughout the body. CLU levels are reduced at the ocular surface in human inflammatory disorders that manifest as severe dry eye, as well as in a preclinical mouse model for desiccating stress that mimics dry eye. Using this mouse model, we show here that CLU prevents and ameliorates ocular surface barrier disruption by a remarkable sealing mechanism dependent on attainment of a critical all-or-none concentration. When the CLU level drops below the critical all-or-none threshold, the barrier becomes vulnerable to desiccating stress. CLU binds selectively to the ocular surface subjected to desiccating stress *in vivo*, and *in vitro* to the galectin LGALS3, a key barrier component. Positioned in this way, CLU not only physically seals the ocular surface barrier, but it also protects the barrier cells and prevents further damage to barrier structure. These findings define a fundamentally new mechanism for ocular surface protection and suggest CLU as a biotherapeutic for dry eye.

## Introduction

The ocular surface is directly exposed to the outside environment, where it is subject to desiccation and interaction with noxious agents, thus it must function as a barrier to protect the underlying tissue [1]. Membrane-associated mucins project from the apical cell layer of the corneal and conjunctival epithelia into the tear film, where they bind multiple oligomers of the lectin LGALS3 to form a highly organized glycocalyx, creating the transcellular barrier [2, 3]. In addition, tight junctions seal the space between adjacent cells to create the paracellular barrier [4]. The barriers appear to be functionally linked via the cytoskeleton [5].

Ocular surface barrier disruption is a sign of dry eye, a disorder caused by inadequate hydration by the tears, which results in discomfort, affects quality of vision, and can cause blindness [6]. Dry eye affects ~5 million people over the age of 50 in the USA (especially women) and almost 15% of the population at all ages, comprising upwards of 30–40 million people [7]. In all forms of dry eye, reduced tear flow and/or increased evaporation leads to tear hyperosmolarity, initiating the vicious circle of dry eye pathology. Hyperosmolarity induces inflammatory cascade activation [8–10], promotes apoptosis [11–13], and stimulates expression and activity of matrix metalloproteinases (MMPs) [14, 15], leading to ocular surface barrier disruption [16]. Disruption of the ocular surface barrier is assessed clinically by measuring uptake of water-soluble dyes such as rose-bengal, lissamine green or fluorescein, which occurs in a distinctive punctate pattern in dry eye [17, 18]. The normal ocular surface exhibits variable levels of dye uptake, possibly reflecting the natural processes of cellular desquamation and shedding of mucin ectodomains [1, 18, 19]. Higher levels of dye uptake are diagnostic of dry eye, however mechanisms are not fully defined [18, 20, 21].

MMP9 is recognized as a causal mediator of ocular surface barrier disruption due to desiccating stress in both mice [14, 15], and humans [22]. To help generate hypotheses about mechanisms of dry eye, we performed a yeast two-hybrid screen for corneal proteins that interact with MMP9 [23]. A single candidate was validated: clusterin (CLU). Functional studies revealed that CLU is a potent inhibitor of MMP9 enzymatic activity, as well as activity of other MMPs. When CLU was added to confluent epithelial cell cultures treated with MMP9, tight junctions were protected against MMP9 proteolysis [23].

Human CLU is secreted as a 62-kDa glycoprotein (with an apparent mass of 70–80 kDa as evaluated by denaturing SDS-PAGE) composed of two disulfide-bonded polypeptide chains derived from proteolytic cleavage of an intracellular precursor [24]. With three sites for N-linked glycosylation on each chain, secreted CLU is 17–27% N-linked carbohydrate by weight [25]. Three long natively disordered regions linked to amphipathic helices form a dynamic, molten globule-like binding site, providing the ability to interact with a variety of molecules [26]. Also known as apolipoprotein J or ApoJ, CLU associates with discrete subclasses of high-density lipoproteins [27]. CLU is cytoprotective [28, 29] and anti-inflammatory [30], and it also functions as an extracellular molecular chaperone, acting to maintain proteostasis by inhibiting the aggregation of stress-induced misfolded proteins and facilitating their clearance from extracellular fluids [31, 32]. Consistent with this, the only known phenotype of CLU knockout mice maintained under unstressed conditions is the gradual accumulation of insoluble protein deposits in the kidney [33]. On the other hand, CLU knockout mice exhibit distinct phenotypes when conditions are created to model inflammatory diseases [30, 34].

CLU is found in bodily fluids and is expressed prominently by epithelia at fluid-tissue interfaces [35, 36]. In the context of its known properties, this expression pattern suggests that CLU protects barrier cells from the environment. With regard to the ocular surface-tear interface, CLU was identified as the most abundant transcript in the human corneal epithelium [37]. CLU is expressed in the apical corneal epithelial cell layers in both human [38] and mouse [23], and has also been identified in human tears [39–41]. Expression of CLU in the ocular surface epithelia is dramatically reduced in human inflammatory disorders that manifest as severe dry eye [38]. Similarly, we showed recently that both CLU protein and mRNA levels in the ocular surface epithelia are reduced by ~30% when desiccating stress is induced in a preclinical mouse model for dry eye [23]. In addition, a striking reduction of CLU expression was observed in cultured human corneal epithelial cells treated with inflammatory mediators [23]. Collectively, these results suggest that down-regulation of CLU expression at the ocular surface subjected to desiccating stress in dry eye is due to activation of the inflammatory cascade.

In this study we investigated the hypothesis that reduced levels of CLU result in vulnerability to barrier disruption using the preclinical mouse model.

## Materials and Methods

### Proteins and antibodies

HUGO nomenclature is used for genes and their products, unless otherwise indicated. The secreted form of recombinant human CLU (rhCLU) and recombinant mouse CLU (rmCLU), both of which contain a polyhistidine-tag (His6 tag) at the C-terminus, were purchased from R&D Systems (Minneapolis, MN). These proteins are expressed in mammalian cells and are fully glycosylated and processed, closely modeling secreted CLU expressed *in vivo*. Natural secreted plasma CLU (pCLU) purified from human serum was purchased from ProsPec (Ness-Ziona, Israel). Bovine serum albumin (BSA) was purchased from R&D Systems. The cytokine TNFA was purchased from Sigma (St. Louis, MO). Anti-CLU (sc-6419) and anti-LGALS3 antibodies (sc-23983) were purchased from Santa Cruz Biotech (Santa Cruz, CA). Anti-OCLN (ab168986), anti-ACTB (ab6276), and anti-His6 tag (ab18184) antibodies were purchased from Abcam (Cambridge, MA).

### Preclinical mouse model

The University of Southern California’s Institutional Animal Care and Use Committee approved the research protocol for use of mice in this study. Research was conducted in adherence with the Association for Research in Vision and Ophthalmology (ARVO) Statement for the Use of Animals in Ophthalmic and Visual Research.

Wild type C57Bl/6J female mice purchased from Jackson Labs (Bar Harbor, ME) were used for all experiments unless otherwise stated. Mice were housed in a pathogen-free barrier facility at USC and kept at 25±1°C, relative humidity 60%±10%, with alternating 12 h light/dark cycles. Euthanasia was performed using compressed CO_2_ gas, according to the American Veterinary Medical Association Guidelines for the Euthanasia of Animals: 2013 Edition.

Desiccating stress was induced in 6–8 week old mice by the air-draft-plus-scopolamine protocol, as previously described [14]. Briefly, scopolamine hydrobromide (Sigma-Aldrich, St. Louis, MO) (0.5 mg/0.2 ml in PBS) was injected subcutaneously in alternating hindquarters, 4 times/day (7 AM, 10 AM, 1 PM, and 4 PM), to inhibit tear secretion. At the same time, mice were exposed to an air draft for 18 hours/day in a room with 80±1°F and <40% humidity at all times. Standard desiccating stress induction was done for 5 days, otherwise, for the period as indicated.

Delivery of CLU was performed as previously described [8, 14]. Eye drops of CLU or BSA were formulated in PBS vehicle and drops were delivered topically to the unanesthetized mouse eye. The standard treatment protocol was 1 uL/eye, 4 times/day, delivered at the time of scopolamine injection. In some experiments drops were delivered a single time. PBS alone was used as the vehicle control.

Corneal epithelial uptake of clinical fluorescein dye Fluoresoft®-0.35% (Holles Laboratory, Cohasset, MA) was assessed quantitatively using fluorometry, as previously described [14]. In some experiments as noted, Alexa-Fluor-dextran (Molecular Probes, Eugene, OR) was substituted.

### Imaging of fluorescein uptake at the ocular surface

Laser scanning confocal microscopy was used to image the punctate pattern of fluorescein uptake, as described [42]. Mice were euthanized following treatment and whole eyes were extracted. The eyes were immersed in PBS while the optic nerve was detached, following which they were placed anterior side up, on a 0.8% agarose plate (NuSieve® GTG® Agarose, Lonza, Rockland, ME). Whole mount digital images (512 x 512 pixels) were captured with a laser-scanning confocal microscope (LSM 5 Pascal, Zeiss, Thornwood, NY) using a 10X objective. Fluorescent images in the central cornea of the samples were captured in Z-section at 1um intervals by using identical photomultiplier tube gain settings and processed using Zen 2012 software (Zeiss) and ImageJ64 software (http://imagej.nih.gov/ij/). The individual layers of the corneal epithelium were captured utilizing the Z-stack option. This technique allows for the specimen to be scanned from the surface to the basal layer of the epithelium. The Z-stack can then be projected into a flat image representing fluorescein uptake through all layers of the epithelium. The software can also combine the Z-stack images into a three-dimensional (3-D) configuration, generating a cross section that is perpendicular to the apical plane. In this way, penetration of fluorescein into the apical, sub-apical, and basal layers of the epithelium can be evaluated.

### Imaging of CLU binding to the ocular surface and LGALS3 affinity chromatography

CLU binding to the ocular surface was visualized using an indirect immunofluorescent labeling technique and imaged by laser scanning confocal microscopy as described above. Antibody (50 ug) to the His6 tag on rhCLU was labeled with CF™-594 (excitation/emission = 593 nm/614 nm) using a CF dye SE protein labeling kit (Biotium, Hayward, CA). The final labeled antibody was prepared in PBS at 1.7 mg/ml after removal of unincorporated dye molecules. CLU-CF-594-Ab complex (CLU at ~110 ug/mL, which is > threshold concentration) was made before instillation to the ocular surface by incubating CLU (2 uL of 200 μg/ml) and labeled antibody (1.5 uL of 1.7 mg/ml) in the dark for 3 h at room temperature (RT). To each eye, 2 uL of CF-594-Ab alone or complex solution was applied for 15 min before extracting eyes for imaging. As a reference point for CLU binding on the ocular surface, eyes in some experiments were co-treated for 5 min before extraction with a fluorescent lipophilic membrane tracer DiO (1 uL) (3,3'-Dioctadecyloxacarbocyanine Perchlorate, Life Technologies; excitation/emission = 484/501 nm), which was dissolved at 1 ug/ml DMSO.

LGALS3 affinity chromatography was performed as previously described [3]. The CLU present in the various collected fractions was quantified by Western blotting.

### Apoptosis assay and epithelial protein analysis

After 7-day DS with PBS or CLU (1μg/ml) treatments, eyes were frozen in OCT solution and cross-sectioned at 10 um thickness. To detect apoptosis, tissue slides were stained for the terminal deoxynucleotidyl transferase dUTP nick end labeling (TUNEL) using the In Situ Cell Death Detection Kit Fluorescein (Sigma-Aldrich) according to the protocol provided by the company with permeabilisation for 12 min at 37°C, and the fluorescent images were obtained by confocal microscopy.

Protein preparation from epithelial tissue lysates was described previously [23]. Protein extracts from individual eyes in the same treatment group (7 mice/treatment) were pooled. 20 ug of protein/sample was resolved by denaturing SDS-PAGE (12% gel) for Western blotting.

### Cell culture model

Cells of the telomerase-immortalized human corneal limbal epithelial cell (HCLE) line [43] were plated in a 96-well plate and left for 7 days to stratify and differentiate, as previously described [23]. To measure secreted MMP9 produced in response to treatments, cell conditioned media samples were subjected to gelatin zymography and Image J analysis [23].

### Tear CLU quantification

Mouse basal tears were collected in mice by instillation of 2 ul of PBS containing 0.1% BSA into the conjunctival sac of each eye, which was then collected with a glass capillary tube from the tear meniscus in the lateral canthus as described [44]. Samples were pooled from 2 eyes. Tear volume was measured using phenol red–impregnated cotton threads (Zone-Quick; Oasis, Glendora, CA) [45]; results were similar to tear volumes reported previously [46]. CLU was quantified using the Mouse Clusterin Quantikine ELISA kit (R&D Systems), according to the protocol provided by the company, which utilized a standard curve.

### CLU knockout mice

In some experiments, Clu knockout mice on the C57Bl/6J background were used. Heterozygous breeders were purchased from Jackson Labs and bred with C57Bl/6J wild type mice to obtain both heterozygotes and homozygotes on the same background. Genotypes of offspring were confirmed by PCR from genomic DNA isolated from tail tips. The PCR primers were previously described [34].

A morphological evaluation was performed on the unstressed ocular surface of CLU knockout mice of both the heterozygous CLU^+/-^ and homozygous CLU^-/-^ genotypes, comparing to wild type C57Bl/6J mice. First, a hand-held 20-diopter indirect lens was used to examine the ocular surface. The ocular surface of eyes from two different mice of the heterozygous CLU^+/-^ genotype was compared to eyes from two different mice of the wild type genotype. Mice were not anesthetized, nor was any topical anesthetic applied to the ocular surface prior to examination. An ophthalmologist and cornea sub-specialist (MH) performed the evaluation. Similarly, the ocular surface of eyes from three different mice of the homozygous CLU^-/-^ knockout genotype was evaluated.

Next, the ocular surface was evaluated by histologic techniques. Briefly, eyes were fixed in 4% formaldehyde and embedded in paraffin. Sections of 6 um were stained with hematoxylin and eosin or periodic acid-Schiff reagent and photographed with a Nikon Eclipse E400 (Garden City, NY) microscope equipped with a Nikon DXM 1200 digital camera. One eye from each of three different mice was examined from the WT, heterozygous CLU^+/-^ or homozygous CLU^-/-^ genotypes (nine eyes total).

Ocular surface ultrastructure was evaluated by transmission electron microscopy. Briefly, a slit was made at the corneal-scleral margin of the eye, which was then immersed in 2% glutaraldehyde, 2% paraformaldehyde in sodium cacodylate buffer, pH7.4, containing 0.025% (w/v) CaCl2, for 60 min at RT. Anterior segments were separated from the lens and posterior segments and held in fixative overnight before being post-fixed in 1% osmium tetroxide and embedded in EmBed (EMS) resin. Thin sections (70 nm) were post-stained with uranyl acetate and lead citrate, viewed in a JEOL 1200 electron microscope, and photographed with an AMT XR-41 TEM digital camera. One eye from each of three different mice was examined from the WT or homozygous CLU^-/-^ genotypes (six eyes total). An ocular pathologist (GRK) evaluated the images.

### Statistical analyses

Treatment groups (DS+PBS versus DS+CLU or DS+BSA) were compared to controls (non-stressed (NS) versus DS) on the continuous study variables with generalized linear regression models, using an identity link function. In the regression model, a generalized estimating equation approach was used to explicitly incorporate the correlated outcomes between eyes within one animal [47, 48]; an exchangeable correlation structure was used. The independent sample t-test was used to compare cell culture results between groups. Two-sided *P* ≤0.05 was considered statistically significant. Analyses were performed using the Statistical Analysis Software (SAS, Version 9.4).

## Results

### Topical CLU protects the ocular surface subjected to desiccating stress

**Fig 1:**
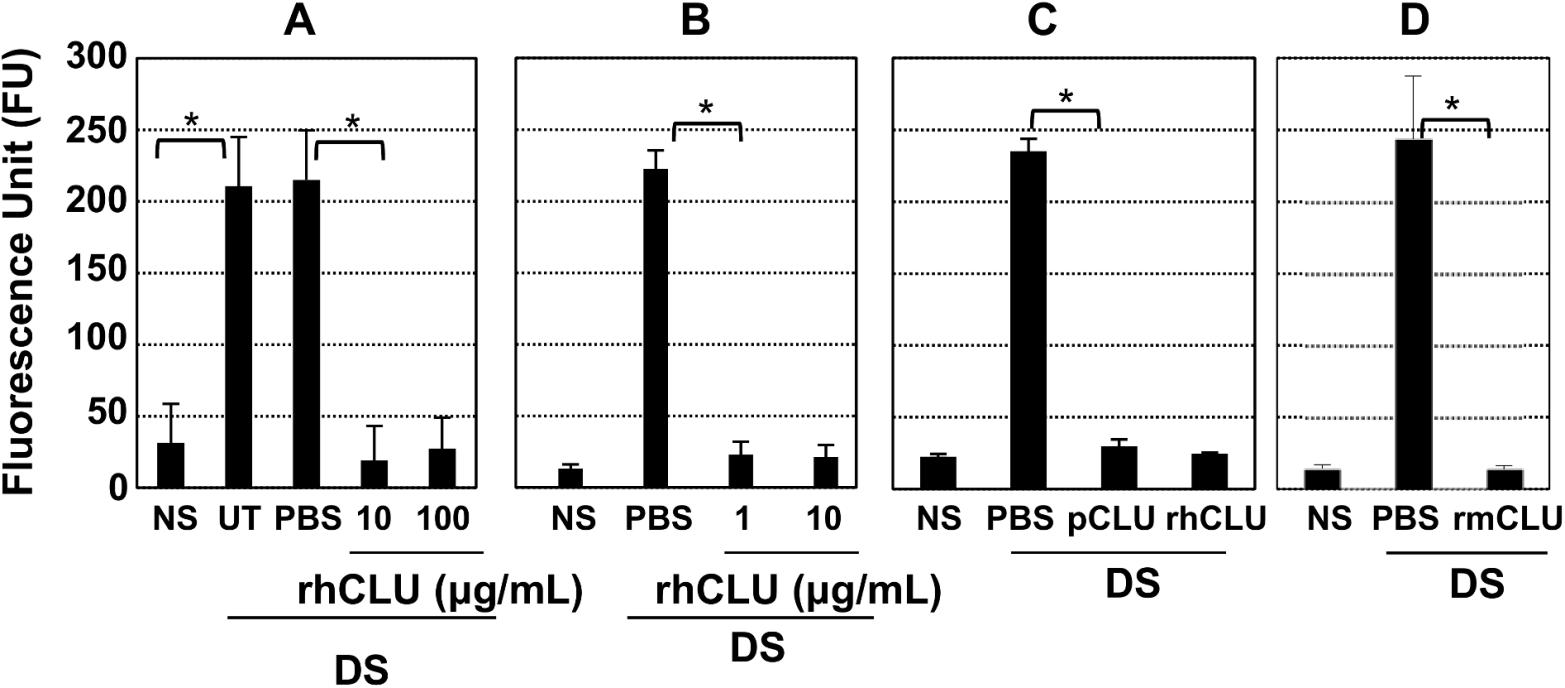
Topical CLU protects the ocular surface barrier against functional disruption by desiccating stress. The standard desiccating stress (DS) protocol was applied, while eyes were left untreated (UT) or treated topically 4 times/day with 1 uL of CLU formulated in PBS, or with PBS control. Non-stressed (NS) mice housed under normal ambient conditions served as a baseline control. After the indicated time period, barrier integrity was assayed by measuring corneal epithelial uptake of fluorescein (FU = Fluorescence Units at 521 nm). Values are expressed as the mean ± SD. **(A)** The desiccating stress (DS) protocol was applied for 5 days while also treating with rhCLU at 10 or 100 ug/mL. *P<0.0001 (n = 9). **(B)** The desiccating stress (DS) protocol was applied for 7 days while also treating with rhCLU at 1 or 10 ug/mL. *P<0.0001 (n = 4). **(C)** The desiccating stress (DS) protocol was applied for 5 days while also treating with human plasma CLU (pCLU) at 2 ug/mL *P<0.0001 (n = 4). (D) The desiccating stress (DS) protocol was applied for 5 days while also treating with recombinant mouse CLU (rmCLU) at 2 ug/mL. *P<0.0001 (n = 4)

To determine whether supplementation with topical CLU could protect against disruption of the ocular surface barrier subjected to desiccating stress, we applied the 5-day desiccating stress protocol to mice, and also treated topically with recombinant human CLU (rhCLU) formulated in PBS, applied 4 times/day at the same time as scopolamine was administered. After 5 days, barrier integrity was quantified by measuring uptake of fluorescein dye. Results were compared to controls treated with PBS vehicle alone. The stressed but untreated (UT) ocular surface served as the control for PBS treatment and non-stressed (NS) eyes served as the baseline control. Since CLU concentration in human serum was known to be in the range of 100±50 ug/mL [49], we used 10 or 100 ug/mL of rhCLU for our first experiments (Fig 1A). Dye uptake in stressed eyes treated with PBS alone was ~8-fold greater than NS counterparts. In contrast, dye uptake in eyes that were stressed, while also being treated with CLU at 10 or 100 ug/mL, was similar to that of NS counterparts, indicating complete protection against barrier disruption. We performed a second set of experiments using a 7-day desiccating stress protocol and rhCLU concentrations of 1 and 10 ug/mL. Again we observed nearly complete protection against barrier disruption as measured by dye uptake at both concentrations (Fig 1B). We performed a similar experiment using a 5-day desiccating stress protocol, but using human plasma CLU (pCLU) (Fig 1C) or recombinant mouse CLU (rmCLU) (Fig 1D) to rule out the possibility that the results might be unique to rhCLU. Treatment with 2 ug/mL of pCLU or rmCLU consistently protected against barrier disruption as measured by fluorescein uptake, to the same extent as rhCLU at 2 ug/mL, and was comparable to NS controls.

### Topical CLU protects the ocular surface in an all-or-none response

**Fig 2:**
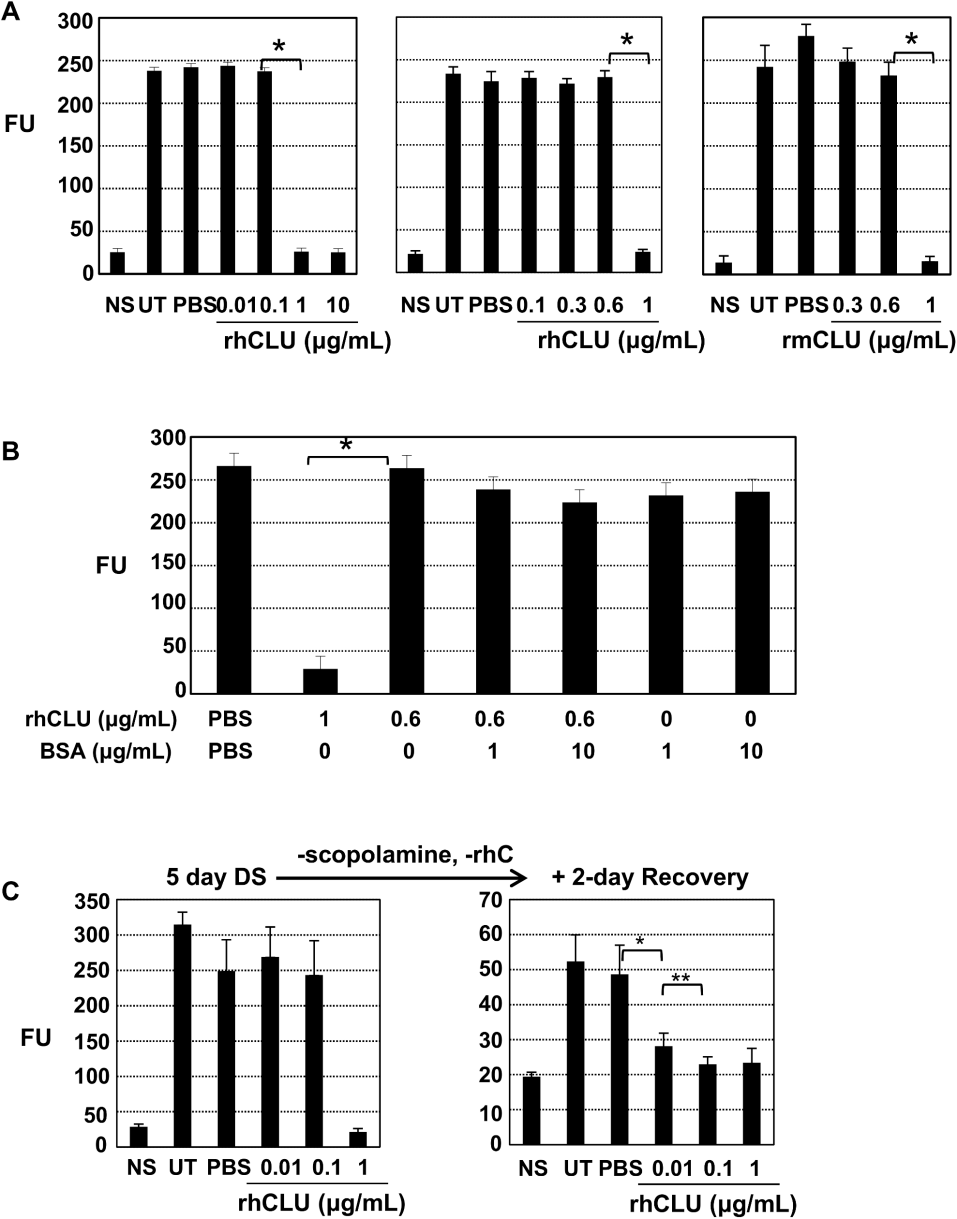
Topical CLU protects the ocular surface barrier via an all-or-none mechanism. The standard desiccating stress (DS) protocol was applied, while eyes were left untreated (UT) or treated topically 4 times/day with 1 uL of CLU formulated in PBS, or with PBS control. Non-stressed (NS) mice housed under normal ambient conditions served as a baseline control. After the indicated time period, barrier integrity was assayed by measuring corneal epithelial uptake of fluorescein (FU = Fluorescence Units at 521 nm). Values are expressed as the mean ± SD. **(A) Dose response experiment.** The desiccating stress (DS) protocol was applied for 5 days while also treating with **(Left)** recombinant human CLU (rhCLU) at the indicated 10-fold dilutions (n = 6), **(Middle)** recombinant human CLU (rhCLU) at 0.1, 0.3, 0.6, or 1 ug/mL (n = 6), or **(Right)** recombinant mouse CLU (rmCLU) at 0.3, 0.6, and 1 ug/mL (n = 4). *P<0.0001. **(B) Experiment comparing CLU with BSA.** The desiccating stress (DS) protocol was applied for 5 days while also treating with recombinant human CLU (rhCLU) and BSA, individually or in combination, as indicated. *P<0.0001 (n = 4)**. (C) Stress reduction experiment.** The standard desiccating stress (DS) protocol was applied for 5 days while eyes were also treated with recombinant human CLU (rhCLU) at 0.01, 0.1, and 1 ug/mL. Using a subset (n = 4) of each treatment group the effect of each rhCLU dose on integrity of the ocular surface barrier was confirmed by the fluorescein uptake test at day 5. Then the rest of the mice in each treatment group were subjected for two more days to a more moderate desiccating stress by continuing with the air draft and heat, but omitting scopolamine and CLU treatments. The fluorescein uptake test was then performed on these remaining mice. *P = 0.004 (n = 4); **P = 0.05 (n = 4)

To determine a dose-response for barrier protection by CLU, we next applied the 5-day desiccating stress protocol while simultaneously treating the ocular surface with serial 10-fold dilutions of rhCLU. Similar to results of the experiment shown above (Fig 1), treatment with 1 ug/mL or 10 ug/mL almost completely protected against fluorescein uptake. In contrast, lower concentrations had essentially no effect, with values similar to UT and PBS-treated groups (Fig 2A Left). To determine any gradation in activity between 0.1 and 1 ug/mL CLU, we tested CLU concentrations at tight intervals in between these doses (Fig 2A Middle). We observed a transition in effectiveness between 0.6 ug/mL and 1 ug/mL, essentially an all-or-none response. We also tested rmCLU; the dose transition was at exactly the same place, between 0.6 and 1 ug/mL (Fig 2A Right). Next, we tested whether BSA, as an *in vitro* protein stabilizer and as a non-CLU protein also found in serum, could enhance the protective activity of CLU at the low concentration. BSA did not show any significant protective or enhancing effect, alone or with CLU at 0.6 ug/mL, compared with 1 ug/mL of CLU alone (Fig 2B). Use of Alexa-Fluor-dextran in the fluorescein uptake assay, which is more discriminating because of its much larger molecular size [14], gave identical results (data not shown).

To determine whether a dose response effect could be observed at CLU concentrations below the threshold level exhibited by the sharp transition, we changed our experimental conditions. As before, we applied the 5-day desiccating stress protocol while simultaneously treating the ocular surface with rhCLU, but then on day 6 we stopped CLU treatment and discontinued scopolamine injections, but maintained a mild stress by continuing the air draft, elevated temperature and reduced humidity. We then waited an additional two days, following which time we assayed barrier integrity by fluorescein dye uptake (Fig 2C). Disruption of the ocular surface barrier after the 2-day moderate desiccating stress was considerably less than observed when the dye uptake assay was done directly following the 5-day desiccating stress protocol. Interestingly, in this setting, we found that the prior delivery of 0.1 ug/mL CLU, 4 times/day was as effective as 1 ug/mL. Again the result was primarily all-or-none, although we observed a small graded effect between 0.01–0.1 ug/mL, which may reflect the transition between desiccating stress conditions.

These results indicate that topical CLU protects the ocular surface barrier against disruption by desiccating stress in an all-or-none manner at a very precise threshold dose range that is highly reproducible.

### Topical CLU ameliorates pre-existing ocular surface barrier disruption due to desiccating stress

**Fig 3:**
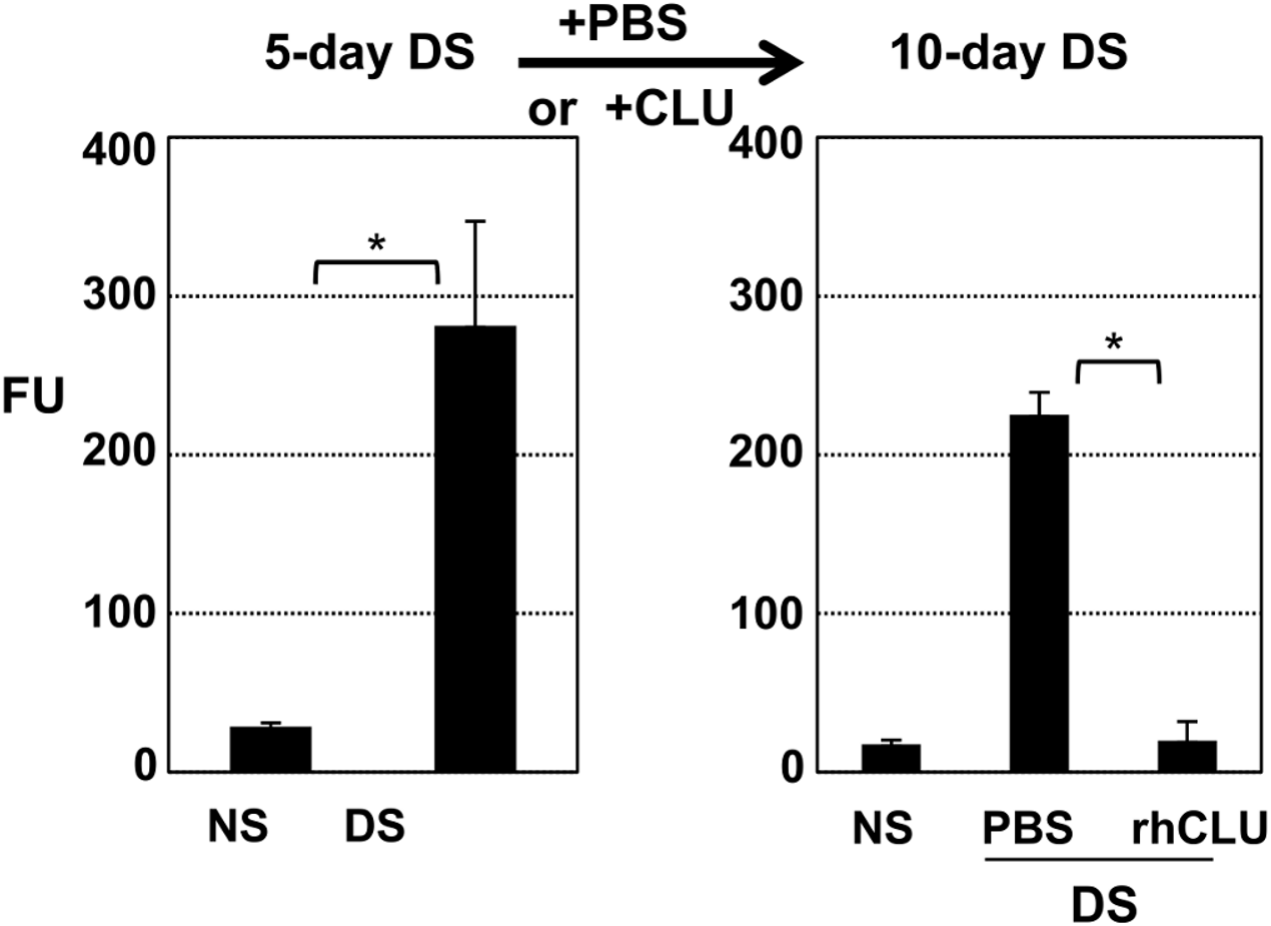
Topical CLU ameliorates pre-existing ocular surface barrier disruption caused by desiccating stress. **(Left).** The standard desiccating stress (DS) protocol was applied for 5-days to create ocular surface disruption. Non-stressed (NS) mice housed under normal ambient conditions served as a baseline control. **(Left)** After the indicated time period, barrier disruption was confirmed by measuring corneal epithelial uptake of fluorescein (FU = Fluorescence Units at 521 nm) in a subset of mice. Values are expressed as the mean ± SD. *p<0.0001 (n = 4). **(Right)** The same desiccating stress (DS) protocol was continued for another 5 days while eyes with desiccating stress were treated topically with 1 uL of recombinant human CLU (rhCLU) formulated in PBS at 2 ug/mL, or with PBS control, 4 times/day. The fluorescein uptake test was then performed on these remaining mice. Values are expressed as the mean ± SD. *p<0.0001(n = 4).

Having clearly demonstrated the preventive effect of CLU in protecting the ocular surface against desiccating stress, we next assessed the potential of CLU to ameliorate pre-existing ocular surface disruption. Representative results are shown in Fig 3. In this experiment, we applied the 5-day desiccating stress protocol, and then treated topically with rhCLU at 2 ug/mL (4 times/day) for another 5 days while maintaining the same desiccating stress protocol. Following this, barrier integrity was assayed. The PBS control showed a high level of dye uptake, ~12X greater than NS controls, but the barrier was essentially intact in CLU treated mice, similar to NS controls.

### Topical CLU directly seals the ocular surface barrier against disruption due to desiccating stress

**Fig 4:**
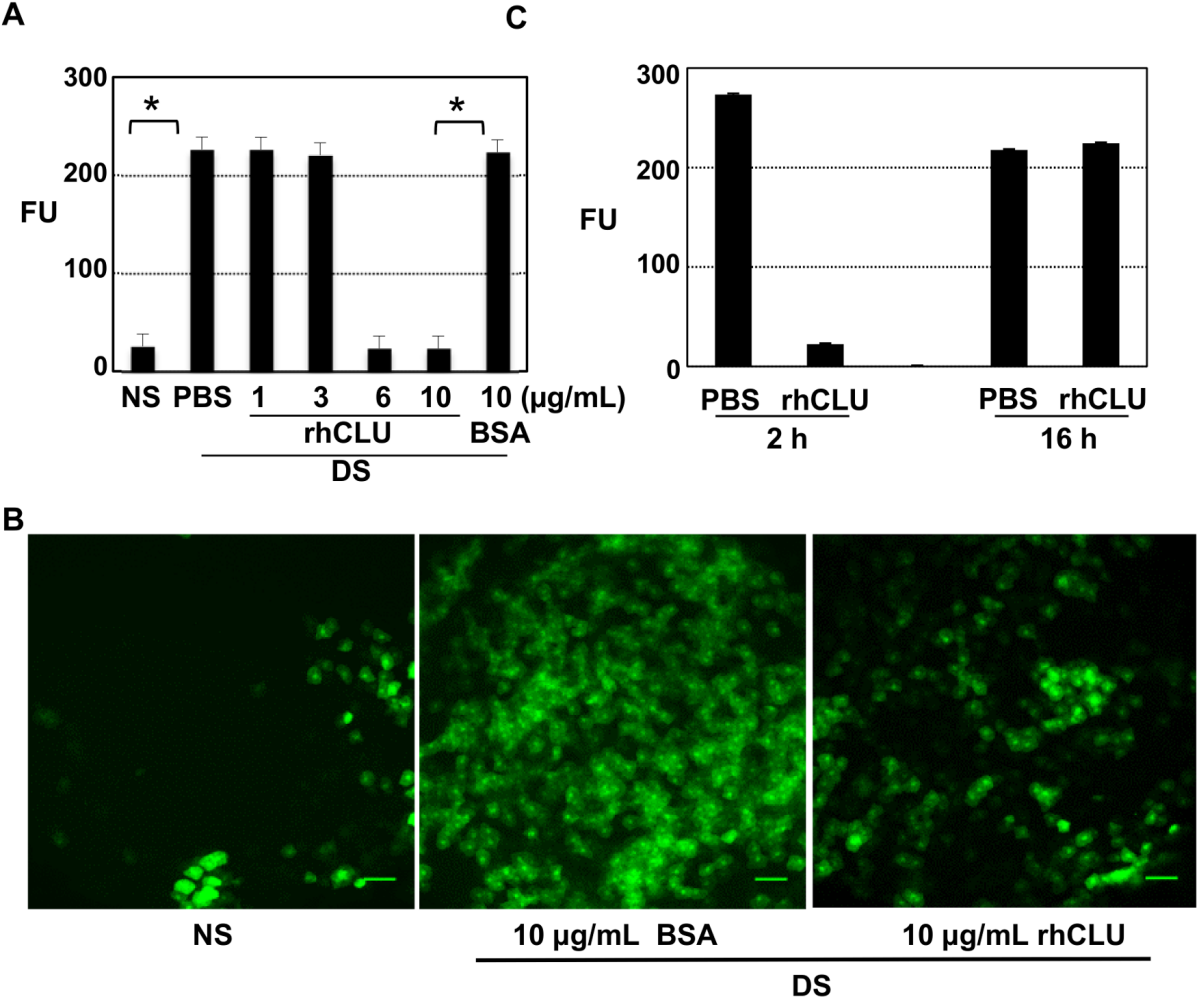
Topical CLU directly seals the ocular surface barrier disrupted by desiccating stress. The standard desiccating stress (DS) protocol was applied for 5-days to create ocular surface disruption. Non-stressed (NS) mice housed under normal ambient conditions served as a baseline control. Eyes with desiccating stress were then treated topically, a single time, with 1 uL of CLU formulated in PBS, 1 uL of BSA formulated in PBS for comparison, or 1 uL of PBS control. Barrier disruption was assayed by measuring corneal epithelial uptake of fluorescein (FU = Fluorescence Units at 521 nm). Values are expressed as the mean ± SD. **(A)** Eyes were treated a single time with recombinant human CLU (rhCLU) at 1, 3, 6 or 10 ug/mL, BSA at 10 ug/mL, or PBS. Fifteen minutes later, the fluorescein uptake test was performed, before there was time for barrier repair to occur. *P<0.0001 (n = 4). **(B)** Images of central cornea from the experiment shown in (A), obtained using laser scanning confocal microscopy at 10X magnification. One representative image out of two independent experiments is shown. Scale bar = 100 um. **(C)** Eyes were treated a single time with recombinant human CLU (rhCLU) at 10 ug/mL (right eyes) or PBS (left eyes). Then the mice were kept further for 2 h or 16 h while continuing with the same desiccating stress protocol. The fluorescein uptake test was performed following the indicated time period to assess the time length of CLU treatment effect. *p<0.0001 (n = 4)

The amelioration results outlined above (Fig 3) suggested that one of the mechanisms of CLU action might be simply to seal areas of barrier damage so that dye can no longer penetrate. To test this idea, we applied the 5-day desiccating stress protocol, and then treated with CLU, but this time assayed for dye uptake within 15 minutes of treatment, giving the ocular surface no time to recover from the more severe stress (Fig 4A). An all-or-none response was observed once again, but the transition point was higher than when CLU was applied 4 times/day. Thus CLU at 6 ug/mL, applied one time, was completely effective in preventing dye uptake, while 3 ug/mL was completely ineffective. Laser scanning confocal microscopy was used to visualize punctate staining and its amelioration (Fig 4B). Eyes of mice subjected to desiccating stress and treated with BSA control showed many punctate spots of the size and shape of cells, similar to UT eyes, while desiccating stress eyes treated with CLU at 10 ug/mL showed far fewer spots, similar to the non-stressed control. In a second set of experiments we sought to determine how long the sealing effect would last. In a time course experiment, the sealing effect was maintained for 2 hours, but was lost by 16 hours (Fig 4C).

### Topical CLU binds selectively to the ocular surface subjected to desiccating stress, and to LGALS3 *in vitro*

**Fig 5:**
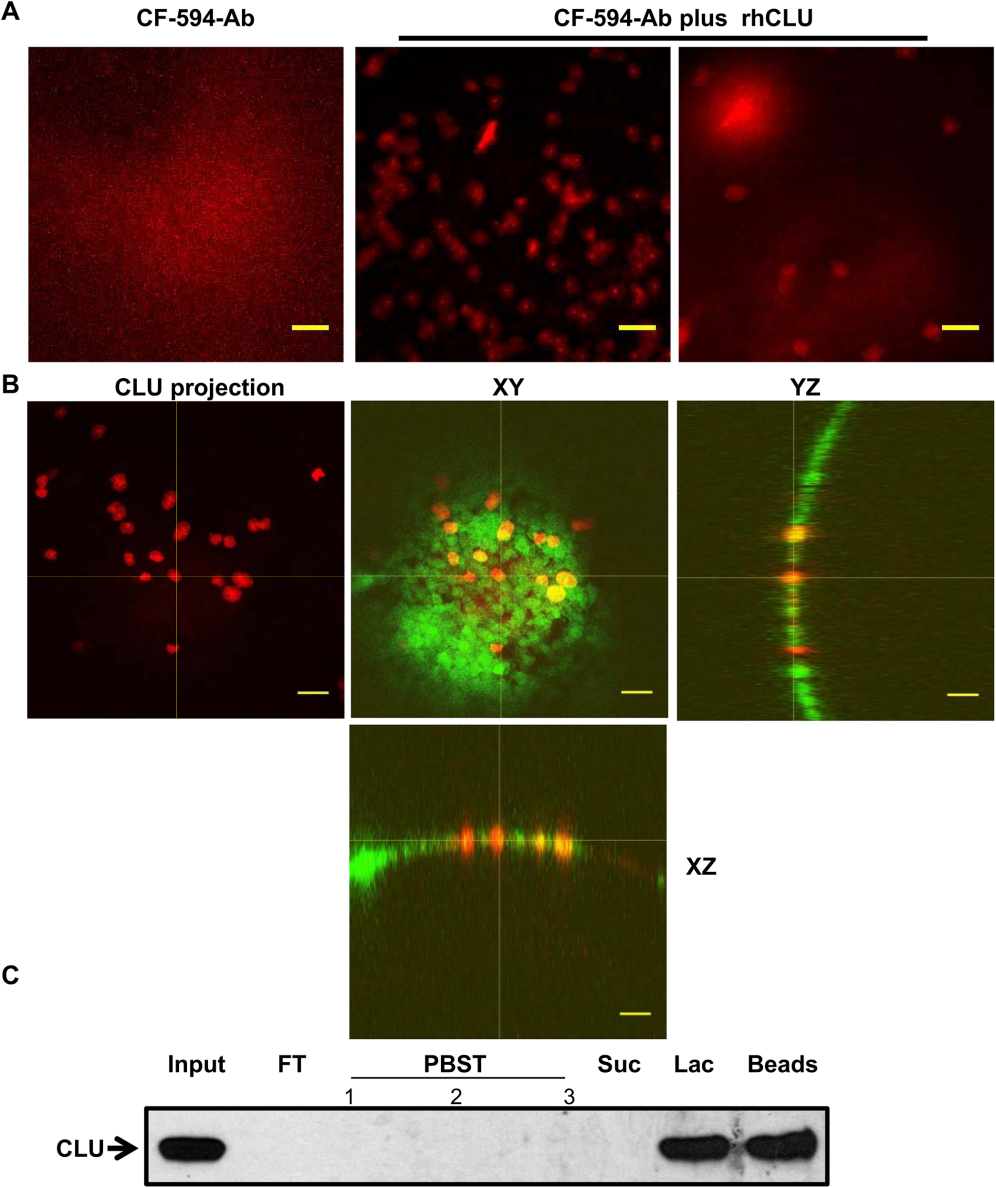
Topical CLU binds selectively to the ocular surface subjected to desiccating stress, and to LGALS3 *in vitro*. **(A)** The standard desiccating stress (DS) protocol was applied for 5-days to create ocular surface disruption. Non-stressed (NS) mice housed under normal ambient conditions were included for comparison. Eyes were treated with CF-594-anti-His antibody that binds to the His tag of recombinant human CLU (rhCLU), or with a complex of the antibody-rhCLU for 15 min, followed by confocal imaging of central cornea. Images were taken at 10X magnification. Scale bar = 100 um. **(B)** A DS eye was treated with a complex of the antibody-rhCLU (red) as in (A), as well as a fluorescent membrane tracer DiO (green). Images were taken at 20X magnification. In the left panel only CLU was projected. The right three panels show one Z-section plane with cross-sections oriented to the XY, YZ, and XZ axes, generated using Image J software. Yellow indicates regions of co-localization of the red and green signal. Scale bar = 100 um. **(C)** LGALS3-Sepharose affinity column chromatography. 1.5 ug rhCLU was applied to a 300 uL LGALS3 affinity column equilibrated in PBS containing 0.1% Triton X-100 (PBST) and the column was washed with PBST. To test sugar-binding specificity, the column was then treated sequentially with a non-competing disaccharide, sucrose (0.1 M), and then a competing disaccharide, 0.1 M lactose, dissolved in PBST. Western blotting was used to quantify CLU in the resulting fractions. Loading of the “Lac” lane represents a 1:10 dilution of the input and the “Beads” lane is a 1:4 dilution of the input, thus ~2.5X more CLU was Lac-eluted than retained on the beads. FT = flow-through; Suc = sucrose; Lac = lactose

To visualize CLU binding to the ocular surface, we used the technique of direct immunostaining with an antibody conjugated to CF-594 dye. To differentiate topically applied rhCLU from endogenous CLU, we took advantage of the C-terminal His tag incorporated into the rhCLU molecule. Representative results are shown in Fig 5A. Eyes subjected to desiccating stress, then treated with CF-594 dye conjugated anti-His antibody alone, showed some diffuse fluorescence over the ocular surface subjected to desiccating stress. However, when the ocular surface of these mice was treated with rhCLU, substantial punctate binding of dye-conjugated antibody to the ocular surface subjected to desiccating stress was observed, indicating the location of direct CLU binding. In contrast, the NS eye showed far less binding. In a second set of experiments, the fluorescent lipophilic membrane tracer DiO was used to delineate individual cells. Representative results are shown in Fig 5B. This showed that the CLU “spots” were approximately the size of cells. In some cases, the CLU spots (red) filled the entire area of individual cells marked by the dyed membrane (green), overlapping completely (yellow color). In other cases, CLU spots were clearly separate.

Next we considered what kinds of ocular surface molecules might bind CLU. LGALS3, a key component of the ocular surface barrier, is a member of the galectin class of beta-galactoside-binding proteins. What is known about the glycosyl moiety of CLU is consistent with LGALS3 binding [25, 27]. CLU applied to an LGALS3-sepharose affinity column bound to the beads and was not eluted 0.1 M sucrose, a disaccharide that does not compete with LGALS3 sugar binding, but was mostly eluted with a competitive inhibitor of LGALS3 sugar binding, 0.1 M beta-lactose (Fig 5C). This suggests that CLU binding to LGALS3 is specific for the beta-galactoside-binding function.

### Topical CLU is cytoprotective and proteostatic

**Fig 6:**
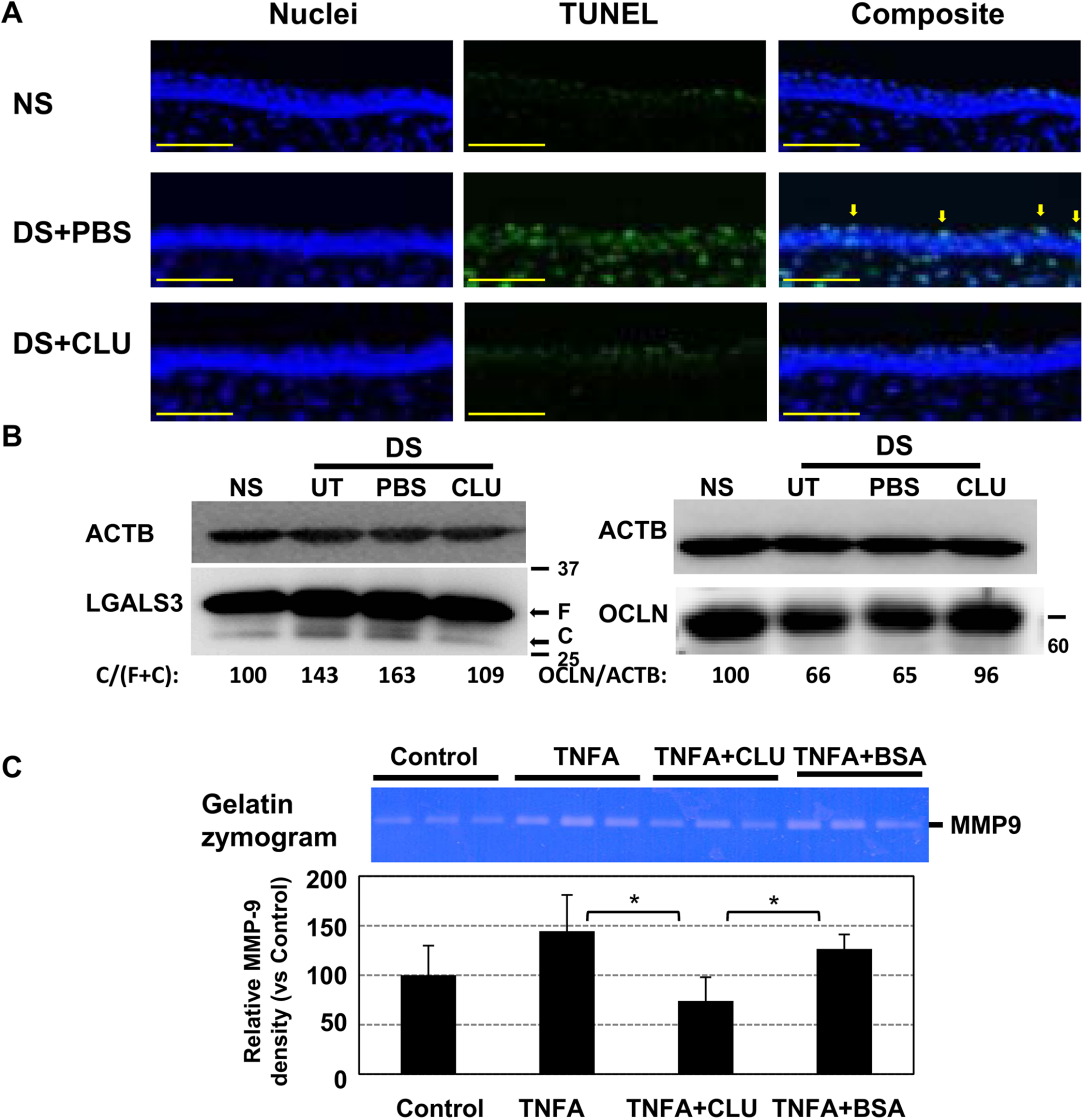
Topical CLU protects the ocular surface barrier against proteolytic damage due to desiccating stress. **(A)** The standard desiccating stress (DS) protocol was applied, while eyes were left untreated (UT) or treated topically, 4 times/day, with 1 uL of recombinant human CLU (rhCLU) formulated in PBS, or with 1 uL of PBS control. Non-stressed (NS) mice housed under normal ambient conditions were included as a control for PBS treatment. At the end of the experiment, eyes were removed and embedded for frozen sectioning at 10-um thickness. TUNEL staining was performed and nuclei were counterstained with DAPI. Images were taken at 20X magnification. Arrows indicate apoptotic cells in the apical ocular surface epithelium of DS+PBS eyes. **(B)** The standard desiccating stress (DS) protocol was applied, while eyes were left untreated (UT) or treated topically, 4 times/day, with 1 uL of recombinant human CLU (rhCLU) formulated in PBS, or with 1 uL of PBS control. Non-stressed (NS) mice housed under normal ambient conditions were included as a control for PBS treatment. Desiccating stress was applied to 7 mice per treatment group for 5 days (OCLN) or 9 days (LGALS3) while treated with PBS or CLU at 1 ug/mL. Then total proteins were extracted from the ocular surface epithelia using TRIzol, pooled among the same treatment groups, and subjected to Western blotting with anti-LGALS3 and anti-OCLN antibodies. The protein band image was obtained by Fuji Doc digital camera. “F” indicates full length LGALS3 protein, and “C” is the cleaved product of LGALS3. A digital image analyzer built into the camera was used to quantify the density of individual protein bands. The relative cleavage of LGALS3 was calculated by ratio of the C over the total (F+C) LGALS3 protein. The relative amount of OCLN was normalized to the loading control (ACTB) in each gel lane. **(C)** Stratified HCLE cells were treated with TNFA (5 ng/mL), alone or with recombinant human CLU (rhCLU) (4 ug/mL) or BSA (40 ug/mL) for 24 h. the conditioned media were subject to gelatin zymography and the developed MMP9 image were analyzed by Image J software. *P<0.05 (n = 3, student’s t-test)

Having demonstrated the capacity of CLU to protect the ocular surface barrier against functional disruption due to desiccating stress, we next tested its capacity to protect the cells and proteins of the barrier against physical damage. First we investigated the cytoprotective activity of topical CLU. Representative results are shown in Fig 6A. Only a few cells at the ocular surface of non-stressed (NS) eyes were positively stained in the TUNEL assay, a measure of DNA damage characteristic of apoptotic cells. In the PBS-treated DS eye, staining of epithelial cells and stroma cells was strikingly increased, consistent with previous observations [11–13, 50]. However, when the ocular surface was treated topically with CLU at the same time as it was subjected to desiccating stress, the level of TUNEL staining remained the same as in non-stressed eyes.

We next investigated protection of ocular surface barrier proteins against desiccating stress. Representative results are shown in Fig 6B. Corneal epithelial lysates were isolated from the eyes of mice maintained under ambient conditions (NS), mice subjected to desiccating stress but otherwise untreated (UT), and mice subjected to desiccating stress while also being treated with rhCLU or the PBS control. We found an increase in a truncated form of LGALS3 after desiccating stress, which suggested proteolysis. Importantly, LGALS3 was protected from truncation in the corneal epithelium of mice treated with topical CLU in PBS, but not in mice treated with PBS alone. Similarly, the amount of the tight junction protein OCLN was reduced in the corneal epithelium of eyes subjected to desiccating stress, but was restored in mice treated with topical CLU in PBS, but not when treated with PBS alone. It should be noted that the area of ocular surface barrier damage is expected to be only a small percentage of the total based on the pattern of punctate fluorescein staining. These findings provide evidence that CLU protects the protein structure of both the transcellular and paracellular barriers at the mouse ocular surface subjected to desiccating stress.

We also examined the effect of CLU on MMP9 expression using a corneal cell culture model, as shown in Fig 6C. Treatment of cells with rhCLU significantly reduced (by ~50%) the stimulatory effects of TNFA on MMP9 expression, but BSA had no effect. These results provide a second possible mechanism for ocular surface barrier protection against proteolysis.

### Causal association between CLU concentration in tears and ocular surface barrier vulnerability

**Fig 7:**
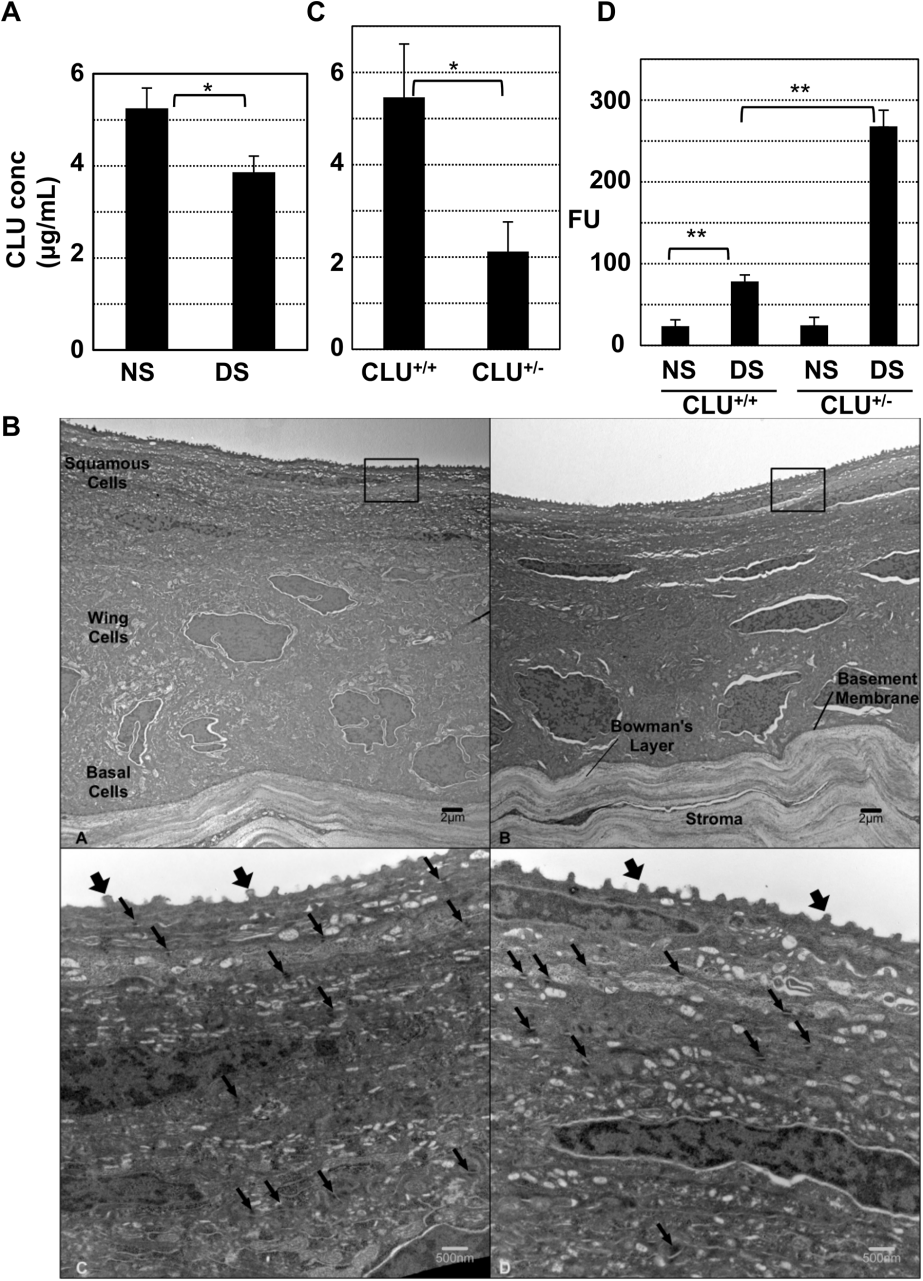
Causal association between endogenous CLU concentration in tears and ocular surface barrier vulnerability. **(A)** Tears were collected from mice housed under normal ambient conditions or after application of the standard desiccating stress (DS) protocol for 5-days, and ELISA was used to measure CLU concentration (*P = 5x10^-8^ n = 6, student’s t-test). **(B)** Representative transmission electron microscopy comparing images of anterior cornea from wild type C57BL6/J mice (A and C) and mice with homozygous CLU^-/-^ knockout on the C57BL6/J background (B and D). In low power (4000x) magnifications (A and B), five layers of epithelial cells divided into squamous, wing, and basal cell regions are visualized along with an intact basement membrane and Bowman's layer in both types of animals. Higher power images (C and D, 20,000x) of similar regions to those boxed in panels A and B show numerous surface microplicae (fat arrows) in both genotypes. Desmosomes (thin arrows) are similar in both frequency and structure. Higher power images (not shown) demonstrate intact adherens junctions in both genotypes. **(C)** Tears from wild type or heterozygous CLU^+/-^ knockout mice kept at ambient conditions were collected and ELISA was used to measure CLU concentration (p = 2.1x10^-5^; n = 7, student’s t-test). **(D)** Wild type mice or heterozygous CLU^+/-^ knockout mice were subjected to the standard desiccating stress protocol, but without scopolamine injection for four weeks and then ocular surface barrier integrity was measured by fluorescein uptake (**p<0.0001, n = 4).

The concentration of endogenous CLU in mouse tears was measured using an ELISA. Representative results are shown in Fig 7A. In this experiment, the mean CLU concentration in tears from mice kept at ambient conditions was 5.2±0.4 ug/mL. This was reduced to 3.7±0.3 ug/mL in tears from mice subjected to the 5-day desiccating stress protocol, an ~30% reduction, similar to what was previously observed in the ocular surface epithelium using this mouse model [23].

CLU knockout mice could be useful for examining the causal relationship between endogenous CLU concentration in tears and ocular surface barrier vulnerability to desiccating stress if the ocular surface is normal under ambient conditions. On gross inspection, eyes of both heterozygous CLU^+/-^ and homozygous CLU^-/-^ knockout mice on the C57BL/6J background appeared anatomically normal. We examined the ocular surface of both of these knockout genotypes more closely using a hand-held 20-diopter indirect lens, and compared to wild type C57BL6/J mice. In all three genotypes, the tear film appeared of similar thickness and the ocular surface appeared smooth and unaffected, with no inflammatory infiltrates apparent. Histological analysis of cross-sections, revealed no differences among genotypes, and periodic acid-Schiff histochemistry revealed similar goblet cell numbers in all genotypes (data not shown).

Ocular surface epithelia examined by transmission electron microscopy revealed no differences between wild type C57Bl/6J and homozygous CLU^-/-^ knockout mice. Representative images are shown in Fig 7B. There was no evidence of squamous metaplasia in the corneal or conjunctival epithelia. Microplicae at the apical cell surface appeared similar in contour and density. Junctional complexes between cells were of similar appearance and numbers. Thus the ocular surface of homozygous CLU^-/-^ knockout mice maintained under ambient conditions appears to be entirely normal, i.e., the same as wild type counterparts.

Next we compared tear CLU concentration in WT and heterozygous CLU^+/-^ mice maintained at ambient conditions. Representative results are shown in Fig 7C. The mean tear CLU concentration in this group of WT mice was 5.5±1.2 ug/mL, while the mean concentration in heterozygous CLU^+/-^ mice was 2.2±0.6 ug/mL. This is an ~50% difference and indicates that the CLU concentration in tears is roughly proportional to the number of gene copies. Significantly, the reduced level of CLU in tears of heterozygous CLU^+/-^ KO mice was less than the level of CLU in tears of WT eyes subjected to desiccating stress. Thus the heterozygous CLU^+/-^ KO genotype can be used to determine whether reduced CLU levels in tears alone results in vulnerability to desiccating stress.

Finally, barrier sensitivity was evaluated in WT and heterozygous CLU^+/-^ KO mice. To facilitate the detection of differences, the mild desiccating stress protocol was used. Thus mice were exposed to air draft at elevated temperature and reduced humidity, but scopolamine injections were omitted (as in Fig 2C). This protocol was continued for 4-weeks, after which time, ocular surface barrier integrity was assayed. Representative results are shown in Fig 7D. Fluorescein uptake in WT eyes was only about 3X higher than NS controls. In contrast, fluorescein uptake in heterozygous CLU^+/-^ KO mice was approximately 10X higher than NS controls. These results demonstrate that reduced CLU in the tears correlates with increased vulnerability of the ocular surface barrier to desiccating stress.

## Discussion

CLU is a homeostatic protein, prominently expressed at fluid-tissue interfaces throughout the body including the ocular surface. Here we report that CLU prevents and ameliorates ocular surface barrier disruption due to desiccating stress by a remarkable sealing mechanism dependent on attainment of a critical concentration in the tears. When tear CLU drops below the critical threshold, the ocular surface barrier becomes vulnerable to disruption. Sealing by CLU involves selective binding to the stressed ocular surface. Positioned in this way, CLU not only physically seals the ocular surface barrier, but it also protects the barrier cells and prevents further damage to barrier structure. These findings provide an answer to the long mystery of CLU’s physiological role at the ocular surface and also identify a fundamentally new mechanism for ocular surface protection.

### Ocular surface sealing

Since the ocular surface barrier of the homozygous CLU^-/-^ KO mouse is intact under ambient conditions, it seems unlikely that CLU is a structural component of the normal barrier, but rather that it serves a protective and surveillance role. This fits with previous reports that CLU knockout mice display a phenotype only when systems are perturbed by application of inflammatory disease models [30, 33, 34]. The selectivity of topical CLU binding for the ocular surface subjected to desiccating stress suggests that CLU seals by binding to areas of barrier disruption. This remains conjectural at this point, as we have not directly demonstrated co-localization with spots of fluorescein uptake, however the punctate character observed for binding of topical CLU at both the normal ocular surface and the ocular surface subjected to desiccating stress is consistent with this idea. Thus we propose that CLU might also act as a “spot weld” at the ocular surface, sealing damage to the barriers where needed.

A previous study suggested that CLU interacts with a lectin-type receptor on liver cells [51] and here we demonstrate CLU interaction with the galectin LGALS3. Galectins are a family of lectin proteins defined by binding specificity for beta-galactoside containing glycans. The main family member at the human ocular surface is LGALS3 (galectin-3) [3, 52, 53]. All galectins have a C-terminal carbohydrate recognition domain, but LGALS3 is unique in also possessing an N-terminal extension with a repeating motif which enables multimer formation [54]. This gives it the capacity to form networks that bridge membrane-associated mucin ectodomains, to organize the ocular surface barrier. MMPs (and likely other proteinases) specifically cleave the multimerization domain from the body of LGALS3, reducing self-association [55–57]. LGALS3 cleavage products are found at the ocular surface and in tears of dry eye patients [58], and we provide evidence here that LGALS3 is cleaved at the mouse ocular surface subjected to desiccating stress. This suggests the possibility that LGALS3 cleavage frees it for interaction with CLU.

CLU sealing may also occur via direct interaction with the plasma membrane of damaged cells. CLU and related apolipoproteins can insert directly into the plasma membrane of cells in the wall of blood vessels [59–61]. This function appears to be due to the special structural features of CLU, in particular the helical amphipathic domains, which confer the properties of a proteinaceous detergent [26]. N-glycosylation sites are located around the disulfide bonds of CLU and may form a scaffold region in clusterin with negatively charged carbohydrates localized to this scaffold. The arms containing the amphipathic helices may extend outward from the scaffold. In this model, CLU resembles a lipid, with the charged head-group being the carbohydrate-covered scaffold of CLU and the hydrophobic tail being the arms. Sealing by CLU may thus be related to the phenomenon of lipid surfactant-mediated “sealing” of plasma membranes damaged by electroporation or other insults, which prevents leakage of fluorescein from preloaded cells [62, 63]. Importantly, insertion of CLU into the vascular wall [59–61] and surfactant-mediated sealing [62, 63] are both cytoprotective. Recently, CLU association with intracellular membranes was also shown to be cytoprotective [64, 65]. The mechanisms of sealing against fluorescein uptake will be very important to define.

### Critical all-or-none threshold

The observation of a critical threshold for all-or-none sealing by topical and endogenous CLU is also quite novel and intriguing. Previous mass spectrometric analyses have indicated that CLU protein is present in human tears [39–41], however the concentration has never been measured in humans or any other species. Here we determine that the concentration of CLU in the tears of mice maintained under ambient conditions is between 5–6 ug/mL. CLU concentration was reduced by ~ 30% (from 5.2 ug/mL to 3.6 ug/mL) in the tears of mice subjected to the 5-day desiccating stress protocol, similar to the percent reduction previously reported in the ocular surface epithelia in this mouse model [23]. Heterozygous CLU^+/-^ KO mice were found to have about half the tear CLU concentration of wild type mice, as would be predicted by the genetic deficiency. This reduction in concentration (to 2.5 ug/mL) results in increased vulnerability to desiccating stress. Adding CLU by topical application corrects this, resealing the barrier.

Our results suggest that the normal concentration of endogenous CLU in tears is above the critical threshold, thus ensuring that the ocular surface barrier remains sealed when subjected to stress. Desiccating stress reduces tear CLU below the threshold, thus making the ocular surface barrier vulnerable to disruption. This means the critical threshold must be somewhere between 5–6 ug/mL and 3.6 ug/mL. Perhaps significantly, this fits within the range of the all-or-none threshold for sealing by a single topical application of CLU (3–6 ug/mL). We envision that topical CLU applied as a 1 uL drop, which is ~30-fold larger than the tear volume [46], would dilute the CLU already present in the tears. Thus, tear CLU contribution to total CLU would seem to be negligible, and the topical threshold for sealing constant, regardless of tear concentration. Enigmatically, a much lower critical threshold is observed when CLU is applied multiple times a day. Perhaps this means we are, in fact, supplementing tear CLU with topical CLU. On the other hand, the mechanism could be much more complex. For example, as noted in the Introduction, CLU has anti-inflammatory properties and thus topical CLU treatment might stimulate a recovery of CLU tear levels over time.

All-or-none responses are seen in many biological processes [66–68] and often involve the assembly of multimeric complexes at a critical concentration [69]. CLU can exist in monomeric or multimeric forms [70, 71] and is found in large complexes in numerous diseases [72–74]. Thus one possible mechanism for the critical threshold effect is that CLU must co-assemble with LGALS3 (and possibly other molecules) into a multimeric complex before it can seal the barrier. Cleavage of LGALS3 alters the carbohydrate binding domain of LGALS3 so that it binds more tightly to glycoconjugates [57], and we show here that LGALS3 binds in a lactose dependent manner to CLU. Significantly, surfactant-mediated sealing of cells occurs only when the surfactant molecules reach a critical concentration in solution, enabling micelle formation. Further studies will be needed to define these important mechanisms.

### Cytoprotection and proteostasis

This is the first time CLU has been demonstrated to be anti-apoptotic at the ocular surface subjected to desiccating stress, however this CLU activity has been well studied in connection with resistance to chemotherapeutics in cancer [28, 29]. Endogenously secreted CLU is re-internalized within the cell by binding to cell surface receptors of the low-density lipoprotein family such as LRP2 (megalin) [75], LPR8, or VLDLR [76], followed by endocytosis. Binding of CLU to LRP2 induces activation of AKT, which phosphorylates Bad [76]. In addition, internalized CLU binds Ku70/Bax complexes, preventing Bax activation [77], and also stabilizes NF-kappaB and IkappaBalpha [78]. Through each of these pathways, internalized CLU increases cell survival and in this way, topical CLU could prevent cells at the ocular surface from entering the apoptotic pathway when subjected to desiccating stress. We must also consider the possibility that CLU’s cytoprotective effect is indirect, a result of its well-known anti-inflammatory activity [30].

An additional and novel means for protecting against apoptosis is suggested by our findings on ocular surface sealing by CLU. As mentioned in the Introduction, mechanisms whereby cells at the ocular surface take up water-soluble dyes are poorly understood. A recent study showed that fluorescein uptake occurs selectively in cultured corneal epithelial cells undergoing apoptosis in response to stress (as opposed to dead cells), suggesting an active transport process [79]. A caveat is that the cultures used in this study were non-confluent, meaning that the tight junction-regulated paracellular barrier would not be fully formed. In addition, the cultures were not stratified, meaning that they would not have expressed cell-associated mucins needed to form the transcellular barrier [23]. Nevertheless, the results suggest the intriguing idea that the immediate sealing of the ocular surface upon topical application of CLU, and the capacity of topical CLU to protect cells from undergoing apoptosis, could be causally linked. Additional studies will be needed to investigate this potentially very important relationship.

Bound at the ocular surface via LGALS3 or other molecules, CLU would be aptly positioned, not only to seal the ocular surface barrier, but also to prevent its further structural damage. The proteostatic effects of CLU as an extracellular molecule chaperone have been well documented [31, 32]. More recently, we showed that CLU is also a potent inhibitor of MMP9 and other MMPs and protects the paracellular barrier against proteolysis by MMP9 *in vitro*. In this study, we provide the first evidence that CLU maintains proteostasis at both the transcellular and the paracellular barriers at the ocular surface subjected to desiccating stress *in vivo*. In addition, using a corneal epithelial cell culture model, we show that CLU reduces MMP9 expression stimulated by the inflammatory cytokine TNFA, providing a second way that CLU might be proteostatic. It should be noted that there are two previously published articles presenting data that CLU stimulates MMP9 expression in cell culture models: leukocytes [80] and tumor cells [81]. We do not consider these results to be conflicting with our own, as CLU activities are often seen to be enigmatic, and may be context-dependent [24]. It is well known that MMP expression can be induced by providing aggregated molecules to stimulate phagocytosis [82], thus the aggregation or multimerization status of CLU may make a difference in its effects on MMP expression.

### Potential of CLU as a novel biotherapeutic for dry eye

Our results demonstrate that topical CLU is remarkably protective of the ocular surface in mice, and can completely reverse the primary sign of dry eye, fluorescein staining. The bioavailability of drugs topically applied to the ocular surface is on the order of 5% or less, due to tear washout effects and the permeability barrier [83, 84], however we show that CLU binds to the ocular surface and remains effective for many hours. These findings, combined with the observed cytoprotective and proteostatic effects of CLU, and considered in context of CLU’s well-characterized anti-inflammatory properties, present a compelling case for developing CLU as a biological therapeutic for dry eye. As a natural homeostatic protein, CLU would be safe and well tolerated, making it an ideal drug. While non-eukaryotic expression systems have been problematic, hrCLU expressed in mammalian cells is full glycosylated, proteolytically processed, and fully functional as a molecular chaperone [85]. Here we show the hrCLU expressed in mammalian cells is functionally indistinguishable from CLU purified from human plasma in protection and sealing of the ocular surface against desiccating stress.

Cyclosporine A (Restasis®, Allergan) is currently the only FDA approved medication for dry eye [86]. The current standard for FDA approval is two studies showing a statistically significant superiority of the drug to its vehicle in relieving both a sign, e.g. fluorescein uptake, and a symptom, e.g., irritation, dryness, gritty feeling and burning [87, 88]. Consistent amelioration of fluorescein uptake has been a difficult criterion for investigational drugs to meet [86–88]. If the all-or-none effect of CLU treatment in mice holds in humans, the “all” part would be an important advantage. Studies in humans are needed to learn whether CLU will also improve symptoms.
